# Supplementary material for: FLVCR1a Controls Cellular Cholesterol Levels through the Regulation of Heme Biosynthesis and Tricarboxylic Acid Cycle Flux in Endothelial Cells
Source: Biomolecules. 2024 Jan 26;14(2):149. doi: 10.3390/biom14020149 (PMC10887198; doi:10.3390/biom14020149)
Supplement: Supplementary file 1 [file biomolecules-14-00149-s001.zip › biomolecules-2812650-Supplementary File S1.pdf]

Supplementary Figure 1

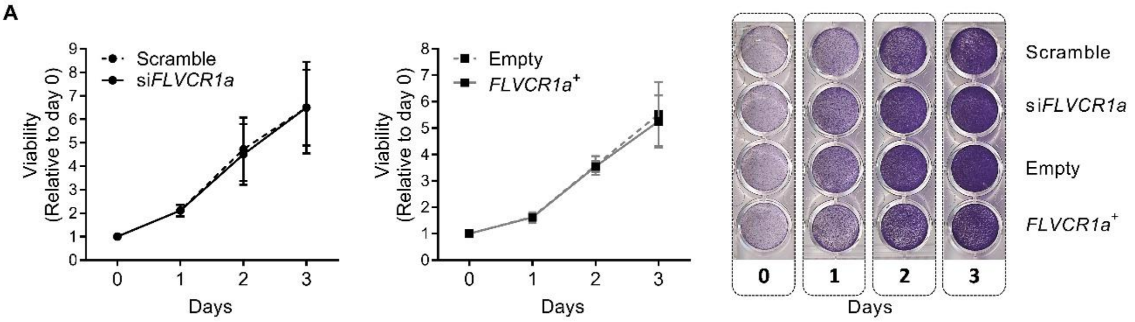

Figure S1. Sk-Hep1 cells viability upon the modulation of FLVCR1a expression

(A) Cell viability was assessed through crystal violet assay. Values are expressed as fold increase at 1, 2, 3 days over day 0 (namely 24h after plating) (left and middle panel). Representative images of cells stained with crystal violet at the analysed time points (right panel). Data are expressed as mean ± SEM (silencing, n=3, three independent experiments; overexpression, n=4, four independent experiments).

si*FLVCR1a*: FLVCR1a-silenced cells; scramble: respective control; *FLVCR1a*$^{+}$: FLVCR1a-overexpressing cells; Empty: respective control.

Supplementary Figure 2

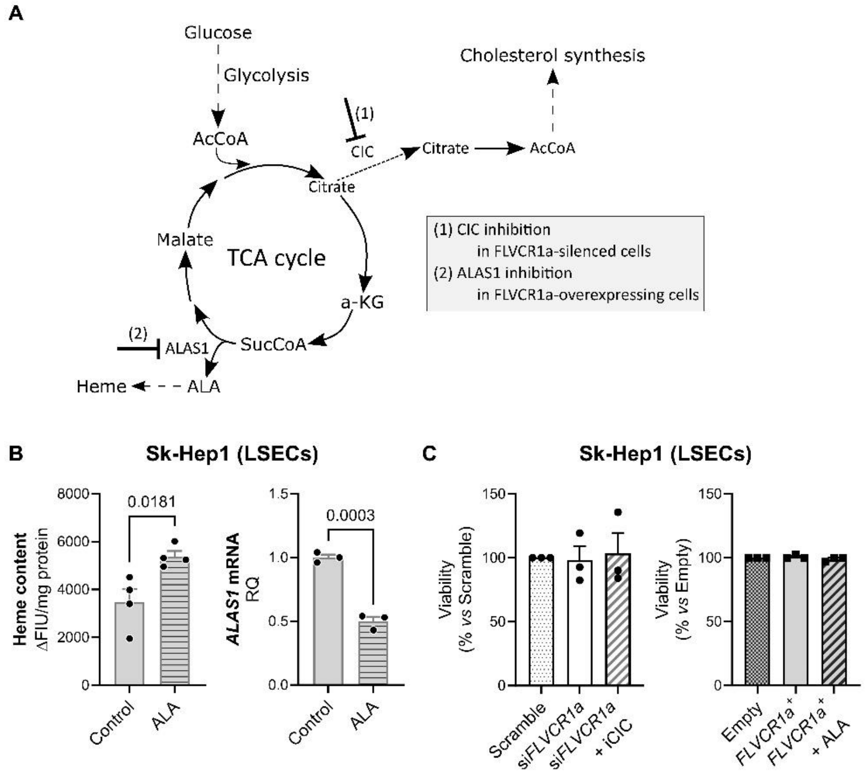

Figure S2. Treatment of Sk-Hep1 cells with iCIC or ALA.

(A) Schematic representation of TCA cycle, heme and cholesterol biosynthetic pathways, as well as of rescue approaches. Citrate export (from mitochondria to cytosol) was inhibited in *FLVCR1a*-silenced cells (1), whereas heme synthesis was inhibited in *FLVCR1a* overexpressing cells (2) by treating the cells with iCIC and ALA, respectively, for 24h. AcCoA, acetyl-CoA; SucCoA, succinyl-CoA; a-KG, α-ketoglutarate; ALAS1, ALA-synthase; CIC, citrate carrier; TCA, tricarboxylic acid; ALA, δ-aminolevulinic acid.

(B) Intracellular heme content (left panel) and mRNA expression levels of *ALAS1* (right panel) in Sk-Hep1 cells upon treatment with ALA. (Left panel) Intracellular heme content is expressed as fluorescence (ΔFIU)/mg protein. (Right panel) Normalization was made using 18S as housekeeping gene. Data are expressed as mean ± SEM of relative quantification using the $2^{-\Delta\Delta Ct}$ method over their respective controls (n=3). Unpaired t-test was performed.

(C) Cell viability upon iCIC and ALA treatment was assessed through crystal violet assay. Values are expressed as percentage (%) compared their respective control cells (scramble or empty). Data are expressed as mean ± SEM (n=3, three independent experiments). One-way ANOVA test was performed.

si*FLVCR1a*: *FLVCR1a*-silenced cells; scramble: respective control; *FLVCR1a*$^{+}$: *FLVCR1a*-overexpressing cells; Empty: respective control; iCIC: mitochondrial citrate carrier inhibitor; ALA: δ-aminolevulinic acid (heme synthesis inhibitor).
