# Supplementary material for: FLVCR1a Controls Cellular Cholesterol Levels through the Regulation of Heme Biosynthesis and Tricarboxylic Acid Cycle Flux in Endothelial Cells
Source: Biomolecules. 2024 Jan 26;14(2):149. doi: 10.3390/biom14020149 (PMC10887198; doi:10.3390/biom14020149)
Supplement: Supplementary file 1 [file biomolecules-14-00149-s001.zip › biomolecules-2812650-Supplementary File S2.pdf]

Figure S1A. Generation of FLVCR1a loss- and gain-of-function models, Sk-Hep1 cells.

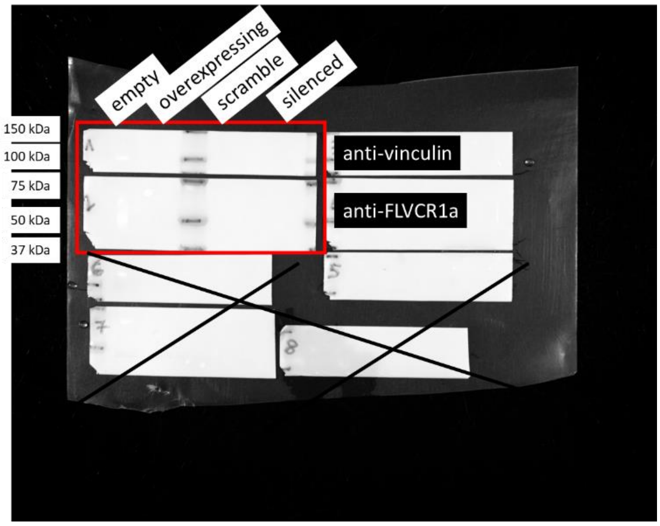

The filter was trimmed, with the section containing high molecular weights probed using the anti-vinculin antibody, while the remaining portion was probed with the anti-FLVCR1a antibody.

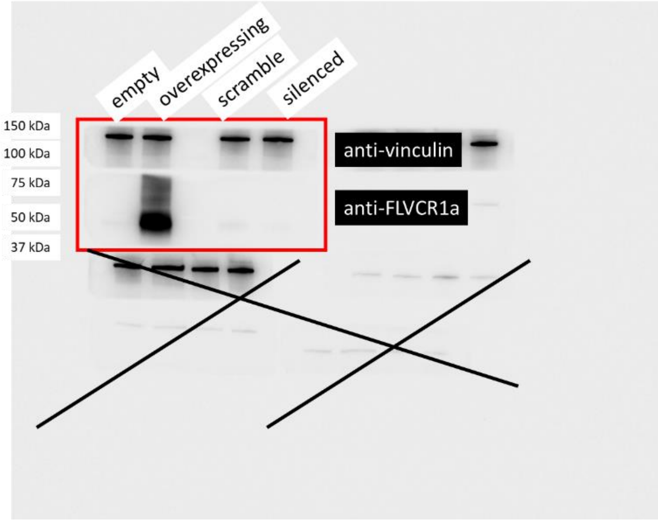

Brief exposure time was employed for the detection of vinculin and the overexpressed FLVCR1a protein.

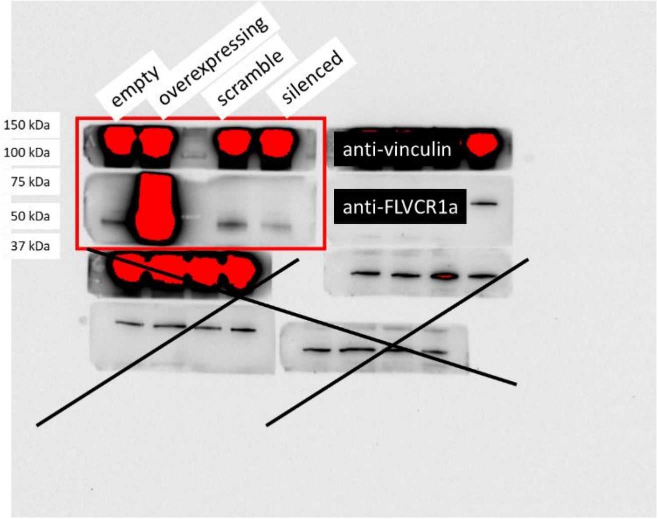

Extended exposure time was utilized to detect the endogenous FLVCR1a and the outcomes of gene silencing.

Figure S1B. Generation of FLVCR1a loss- and gain-of-function models, BTECs.

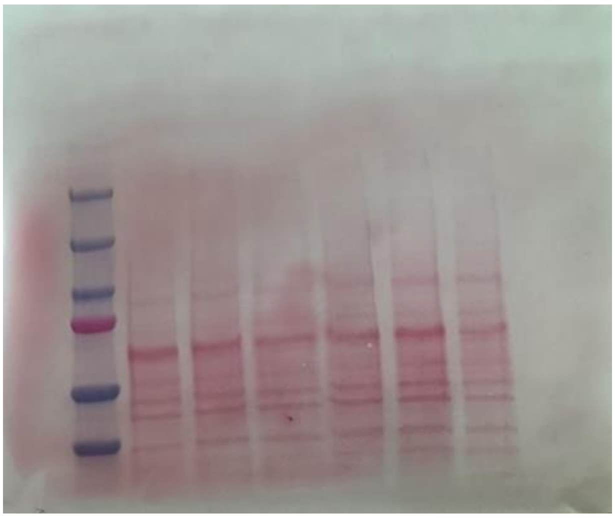

Red Ponceau staining was performed. Lane 1: molecular weight markers, lanes 2-4: control cells, lanes 5-7: Flvcr1a silenced cells. The filter was trimmed, with the high molecular weight region probed using the anti-vinculin antibody, while the other portion was probed with the anti-FLVCR1a antibody.

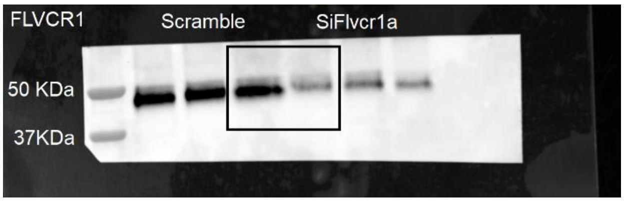

Blot with the anti-FLVCR1a antibody. Lanes 4 and 5 were included in the main text.

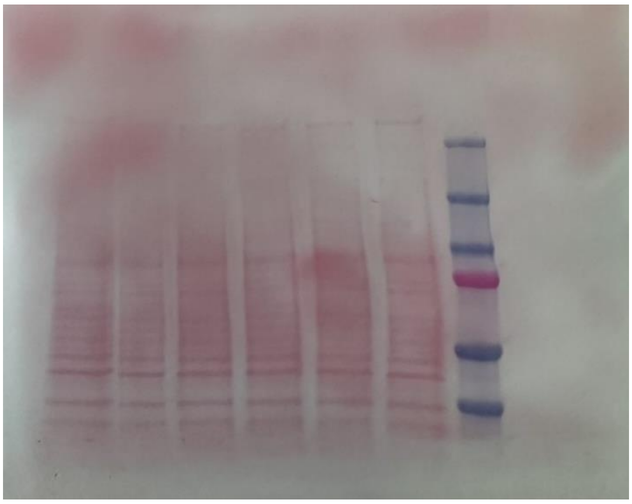

Red Ponceau staining was performed. Lane 1-3: control cells, lanes 4-6: Flvcr1a overexpressing cells, lane 7: molecular weight markers. The filter was trimmed, with the high molecular weight region probed using the anti-vinculin antibody, while the other portion was probed with the anti-FLVCR1a antibody.

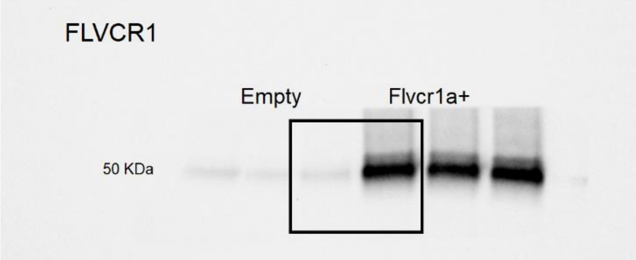

Blot with the anti-FLVCR1a antibody. Lanes 3 and 4 were included in the main text.

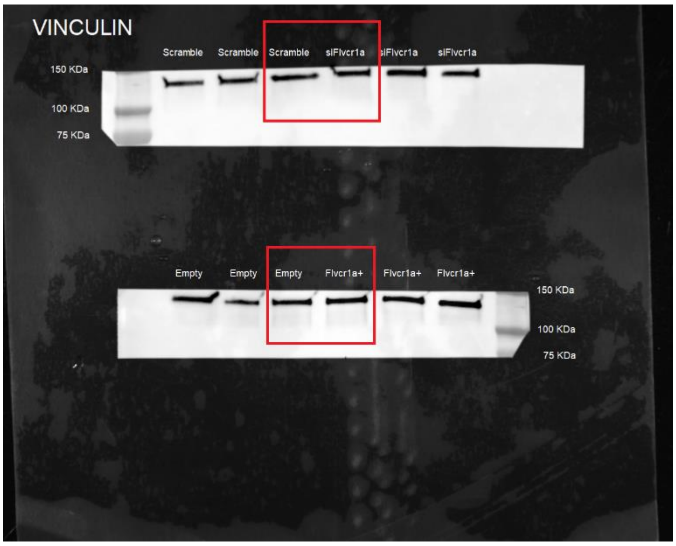

Blot with the anti-vinculin antibody. Top: lanes 4 and 5 were included in the main text. Bottom: lanes 3 and 4 were included in the main text.
